# Supplementary material for: Dynamics of Serum Thymidine Kinase 1 at the First Cycle of Neoadjuvant Chemotherapy Predicts Outcome of Disease in Estrogen-Receptor-Positive Breast Cancer
Source: Cancers (Basel). 2021 Oct 29;13(21):5442. doi: 10.3390/cancers13215442 (PMC8582392; doi:10.3390/cancers13215442)
Supplement: Supplementary file 1 [file cancers-13-05442-s001.zip › cancers-1385182-supplementary.pdf]

Supplementary Materials: Dynamics of Serum Thymidine Kinase 1 at the First Cycle of Neoadjuvant Chemotherapy Predicts Outcome of Disease in Estrogen-Receptor-Positive Breast Cancer

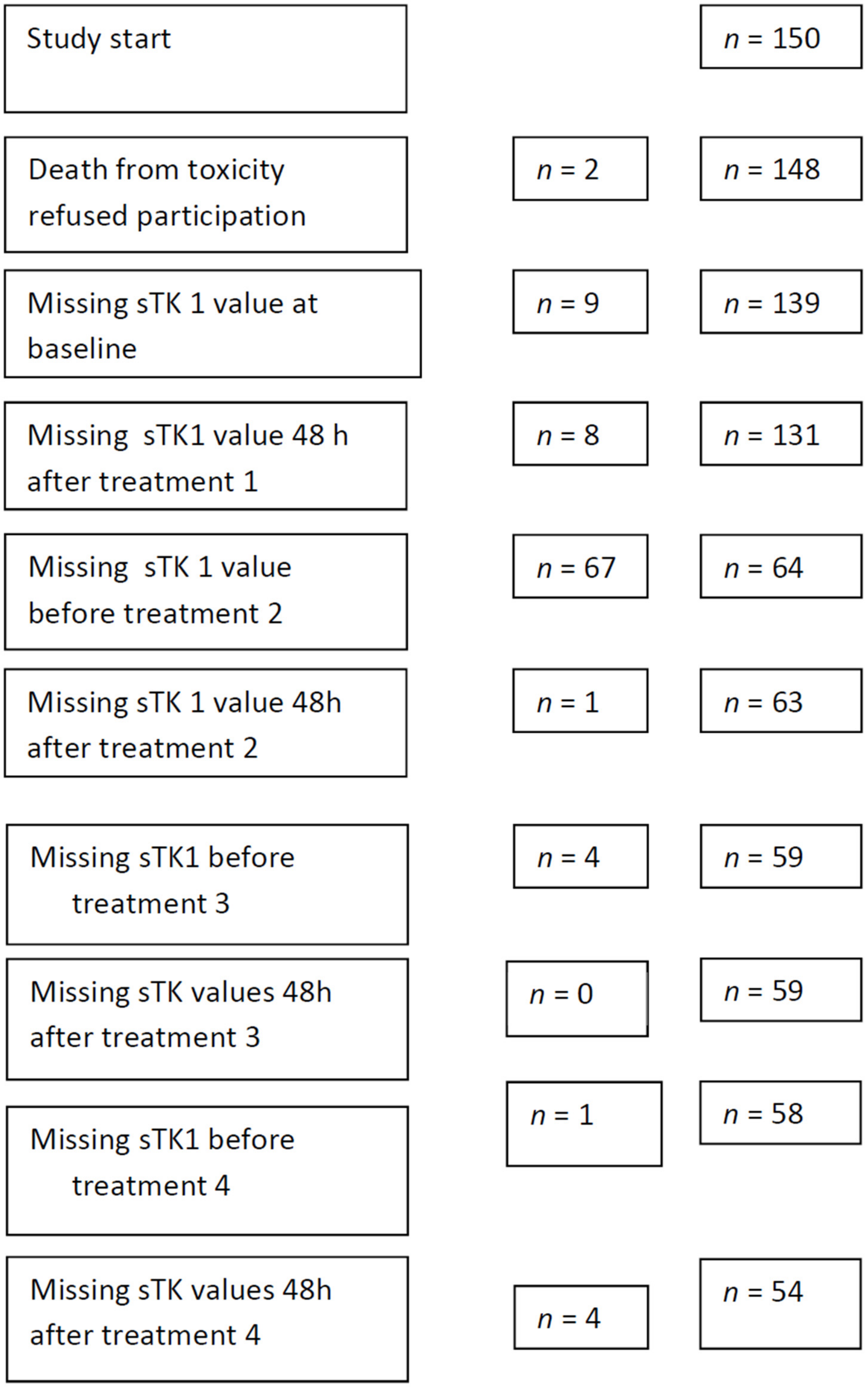

Figure S1. Explanation of missing data among 150 patients with breast cancer, initially recruited for the PROMIX study.

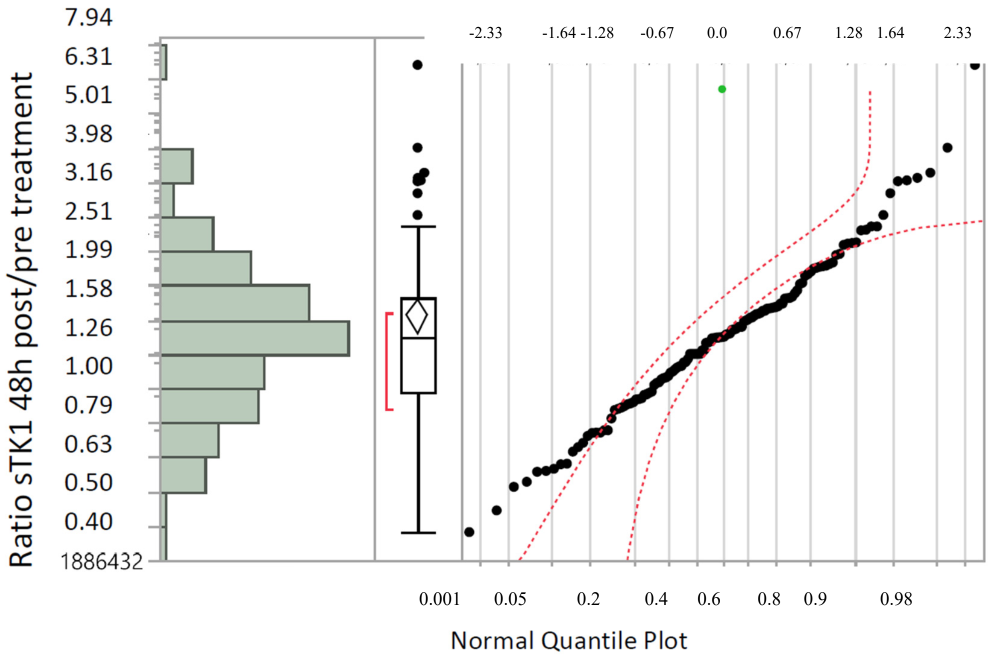

Figure S2. Normal quantile plot of the ratio: serum thymidine kinase 1 concentration (sTk1) 48h post/pre-treatment at cycle 1 in 131 patients with localized breast cancer subjected to neoadjuvant chemotherapy with docetaxel + epirubicin.

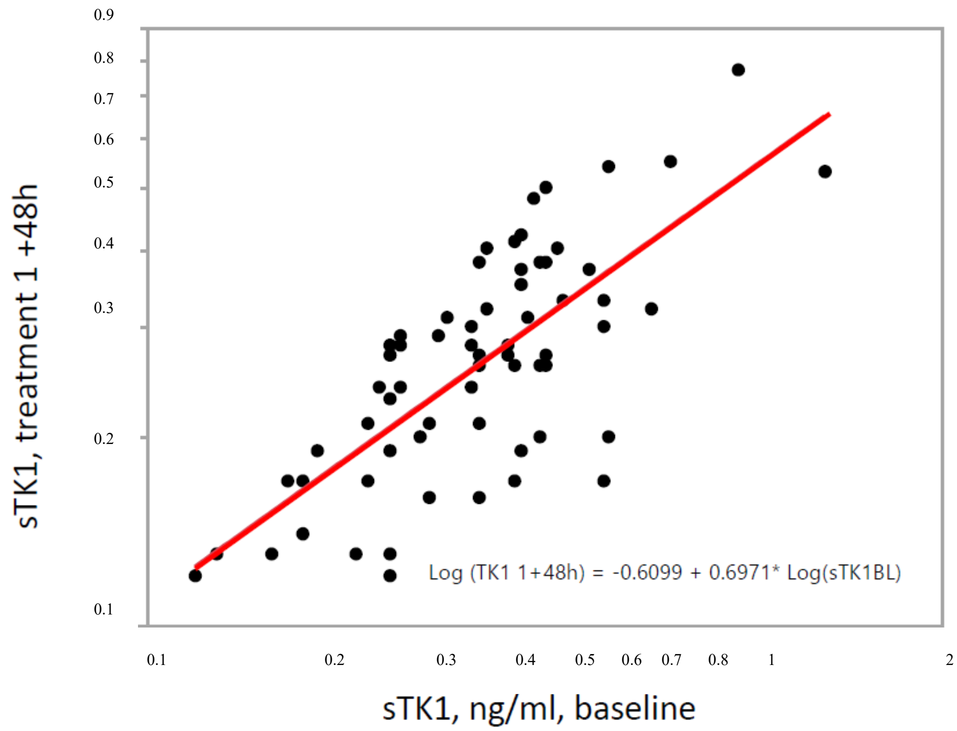

Figure S3. a. Bivariate analysis of serum thymidine kinase 1 concentration (sTk1) before and 48h after treatment 1 in 65 patients with post/pre-treatment ratio <1.12. $r = 0.73$; $p < 0.0001$.

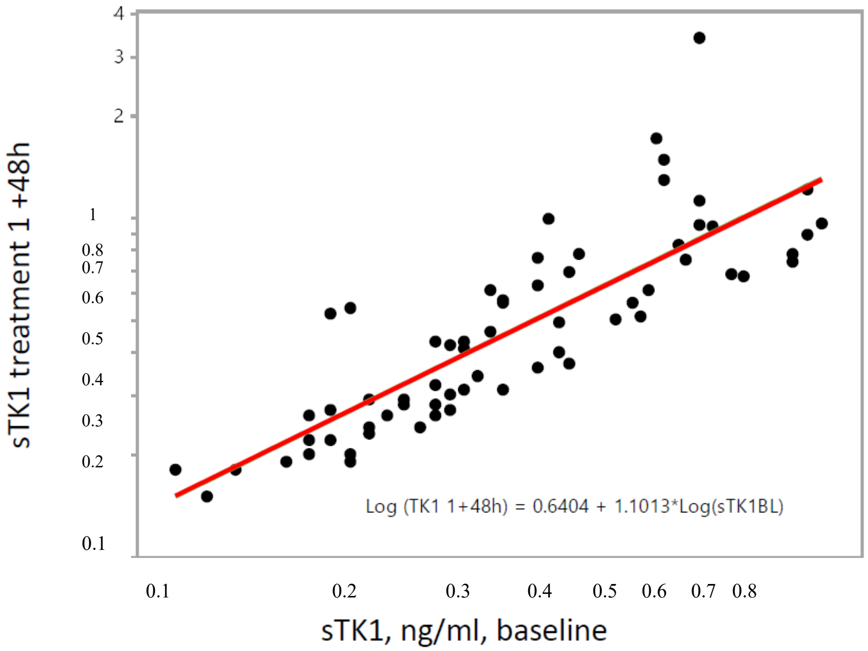

Figure S3. b. Bivariate analysis of serum thymidine kinase 1 concentration (sTk1) before and 48h after treatment 1 in 66 patients with post/pre-treatment ratio >1.12. $r = 0.83$; $p < 0.0001$.
